# Multimodality Treatment of Advanced Non-small Cell Lung Cancer: Where are we with the Evidence?

**DOI:** 10.1007/s40137-018-0202-0

**Published:** 2018-02-08

**Authors:** Christopher M. Jones, Alessandro Brunelli, Matthew E. Callister, Kevin N. Franks

**Affiliations:** 1 Leeds Institute of Cancer & Pathology, Faculty of Medicine & Health, University of Leeds, Leeds, UK; 2 Radiotherapy Research Group, Leeds Cancer Centre, The Leeds Teaching Hospitals NHS Trust, Leeds, UK; 3 School of Molecular & Cellular Biology, Faculty of Biological Sciences, University of Leeds, Leeds, UK; 4 Department of Thoracic Surgery, The Leeds Teaching Hospitals NHS Trust, Leeds, UK; 5 Department of Respiratory Medicine, The Leeds Teaching Hospitals NHS Trust, Leeds, UK

**Keywords:** Lung cancer, Treatment, Radiotherapy, Stereotactic ablative radiotherapy, Chemotherapy, Surgery, Locally advanced, Oligometastatic

## Abstract

**Purpose of review:**

The majority of patients with non-small cell lung cancer (NSCLC) present with advanced disease and overall survival rates are poor. This article outlines the current and outstanding evidence for the use of multimodality treatment in this group of patients, including in combination with an increasing number of treatment options, such as immunotherapy and genotype-targeted small molecule inhibitors.

**Recent findings:**

Optimal therapy for surgically resectable stage III disease remains debatable and currently the choice of treatment reflects each individual patient’s disease characteristics and the expertise and opinion of the thoracic multi-disciplinary team. Evidence for a distinct oligometastatic state in which improved outcomes can be achieved remains minimal and there is as yet no consensus definition for oligometastatic lung cancer. Whilst there is supporting evidence for the aggressive management of isolated metastases, the use of consolidative therapy for multiple metastases remains unproven.

**Summary:**

Evolution of new RT technologies, improved surgical technique and a plethora of interventional-radiology-guided ablative therapies are widening the choice of available treatment modalities to patients with NSCLC. In the setting of resectable locally advanced disease and the oligometastatic state, there is a growing need for randomised comparison of the available treatment modalities to guide both treatment and patient selection.

## Introduction

Lung cancer is the most common malignancy worldwide with over 1.8 million new cases diagnosed each year [1]. Overall survival is poor and only around 15% of patients are alive 5 years after their initial diagnosis [1]. Approximately 80–85% of lung cancers are non-small cell lung cancer (NSCLC), a classification that amongst others encompasses squamous cell carcinoma, adenocarcinoma and large cell, or undifferentiated, carcinoma of the lung. For patients with NSCLC, treatment options vary significantly by disease extent but have considerably evolved across all disease stages as a consequence both of the identification of a raft of targetable, clinically significant molecular aberrations, and of significant innovation in surgery and technical radiotherapy (RT). This article outlines the current and emerging impacts of these advances on the multimodality treatment of advanced NSCLC within the specific settings of locally advanced and oligometastatic disease.

## Early NSCLC: Stages I and II

Effective from January 2017, staging of NSCLC is assessed using the 8th version of the TNM staging system [2]. For the small proportion of patients who present with early (stages I and II) NSCLC, resection remains the gold-standard treatment [3]••, [4]. Overall survival following lobectomy is encouraging; 52–89% of patients with stage I disease and between 33 and 52% with stage II cancer survive 5 years [3••]. Post-operative recurrence is however a concern and the role of adjuvant RT (post-operative radiotherapy; PORT) and chemotherapy has been extensively studied, both in the context of patients with confirmed early NSCLC and for those who are upstaged based on pathology following resection.

There is high-level evidence confirming a lack of benefit from either chemotherapy or PORT in the setting of confirmed stage I disease and neither are routinely recommended [3]••, [5]•, [6, 7]. It has however been suggested that adjuvant chemotherapy may be of benefit in a subset of patients with stage IB tumours exceeding 4 cm in size. Supporting this, the 2008 Lung Adjuvant Cisplatin Evaluation (LACE) meta-analysis pooled individual patient data for 4585 patients from five trials in which adjuvant chemotherapy using a cisplatin backbone had been evaluated [8]. Hazard ratio (HR) for death was 1.4 for stage IA disease but improved to 0.9 in IB disease. Similarly, in a 2008 Cancer & Leukaemia Group B randomised controlled trial (RCT) focussed specifically on IB disease, a significant survival advantage was seen for patients with a tumour size of greater than 4 cm who received doublet chemotherapy consisting of carboplatin and paclitaxel [9].

In contrast, chemotherapy is routinely used post-operatively in patients with nodal disease (i.e. stage IIB or above), where it is thought to confer a survival advantage of around 4–5% [5•]. The use of PORT in these settings is more controversial, particularly in those for whom the cancer has been upstaged following surgery or as a consequence of the pathological identification of N2 nodal disease. Evidence relating to the use of PORT and chemotherapy in stage II and III disease is outlined later in this article.

For patients with early, peripheral disease who are unwilling to undergo or whose medical comorbidity precludes them from surgery, the introduction of high-dose per fraction RT, ‘hypofractionated RT’, has provided an alternative radical treatment option [10]. Highly targeted stereotactic ablative RT (SABR), alternatively known as stereotactic body RT (SBRT), is the preferred option and utilises 4-dimensional CT (4D-CT) planning and hypofractionation to focus a biologically equivalent dose of greater than 100 Gy on the tumour whilst minimising dose to organs-at-risk. Though SABR is now well established in the treatment of stage I NSCLC, the outcomes of prospective comparisons with conventionally fractionated RT and surgery are awaited. In some instances, salvage surgery may be feasible following the use of SABR but a detailed discussion of this is beyond the scope of this article [11].

## Locally Advanced NSCLC: Stage III

Around 70% of patients have advanced cancer at diagnosis. Locally advanced (stage III) disease is an important yet controversial diagnostic subgroup representing 25–30% of all NSCLC diagnoses. Within this cohort, there is significant heterogeneity in both tumour size and the extent of nodal involvement. Consequently, there is no single definitive therapeutic approach and debate continues as to the optimal timing, sequencing and combination of surgery, chemotherapy and radiotherapy across the spectrum of locally advanced disease.

### Sub-classification of Stage III Disease

European guidelines advocate the use of PET-CT and contrast-enhanced brain MRI to evaluate nodal involvement and exclude both extra-thoracic and intracranial metastases during the work-up of stage III NSCLC [3]. Cases in which a tumour of over 5–7 cm in size is associated with N1 disease, or a tumour of any size has metastasised to contralateral nodes (N3) or ipsilateral mediastinal or subcarinal nodes (N2), are classified as locally advanced. Stage III disease was subdivided by the 7th Edition of the TNM system into two broad subcategories, IIIA and IIIB [12]. Stage IIIA disease broadly described a large tumour size or significant nodal disease confined to the affected lung or ipsilateral mediastinum. Stage IIIB disease was then characterised by contralateral nodal (i.e. N3) involvement, regardless of tumour size. In contrast, the 8th TNM staging system retains only ipsilateral disease in stage IIIA but grades large tumours greater than 5 cm (T3 or T4) with ipsilateral nodal involvement (N1 or N2) or smaller (T1 or T2) tumours with contralateral nodal involvement (N3) as stage IIIB. The addition of a third stage of IIIC is used to group large (T3 and T4) tumour size with contralateral nodal involvement but no distant metastatic disease.

This reclassification of locally advanced disease results from proposals made by the International Association for the Study of Lung Cancer (IASLC) International Staging Project based on analysis of just over 70,000 reported cases of NSCLC from across the globe [13••]. When these stages were retrospectively applied to the studied cohort, respective survival was found to significantly differ at 41, 24 and 12% for stage IIIA, IIIB and IIIC diseases [13••]. At present, this revised classification is of limited use to treatment selection. It is however likely to contribute to development of the evidence base for locally advanced disease by establishing subgroups in which disease heterogeneity is limited, and in which novel treatments and combinations of treatment modalities can be prospectively assessed.

### The Multimodality Treatment of Potentially Resectable Stage III Disease

A more clinically useful distinction is that between resectable and non-resectable NSCLC. Resectable disease typically includes T4N0 tumours for which complete (R0) resection is considered by a multi-disciplinary team (MDT) to be feasible, or N2 disease with single nodal station involvement. It additionally includes those patients initially considered to have early disease but who are upstaged following the operative recognition of N2 disease. Unresectable disease includes IIIA NSCLC with bulky or multiple mediastinal lymph node involvement or IIIB disease that is T4 and has mediastinal node involvement.

### Upstaged Disease: Primary Surgery Followed by Adjuvant Treatment

Despite the now widespread use of sophisticated staging investigations, a proportion of patients will only be identified to have N2 disease, and therefore be upstaged from stage I or II disease to IIIA, intra-operatively. Recurrence following surgery remains high and a more advanced stage is associated with increased propensity to recurrence and a shorter disease-free interval. In some analyses, a more advanced stage has been linked with a 30–90% increased risk of mortality, the likelihood of which correlates with either incomplete macroscopic resection or the presence of micrometastases [14]. For patients for whom N2 disease is identified incidentally, adjuvant therapy can improve survival.

There is strong evidence for the use of adjuvant chemotherapy and it is routinely recommended [3••]. A 2016 Cochrane review evaluated the effect of PORT on survival and recurrence in patients with NSCLC that had been completely resected [15••]. Fourteen trials were included, providing 2343 participants who were subsequently analysed in a quantitative meta-analysis using data derived from individual participants of the included trials. This identified adverse outcomes following PORT, with a hazard ratio of 1.18 and a reduction in overall survival at 2 years of 5–53%. The authors conclude that PORT should not be routinely recommended, though the studied patients did not benefit from modern RT techniques, which can reduce toxicity and positively impact on survival. The international multicentre phase III Lung ART trial (NCT00410683) is ongoing and will compare disease-free survival (DFS) following PORT using modern RT techniques to no PORT in patients with N2 disease post-surgical resection.

### Resectable IIIA/N2 Disease

In patients with resectable N2 (i.e. IIIA) disease identified via pre-operative staging, available multimodality treatment options include definitive surgery with pre-operative or post-operative chemotherapy alone or with PORT or definitive chemoradiotherapy (dCRT). None of these approaches is yet recognised to confer a statistically significant overall survival advantage and the involvement of an experienced MDT in treatment selection is vital to determining the optimal treatment for this heterogeneous group of patients.

The current perceived treatment equipoise is based on a number of prospective trials in which no significant difference in overall survival has been seen. In the Intergroup trial, induction CRT followed by surgery improved progression-free survival when compared with dCRT but failed to impact on overall survival [16•]. Pneumonectomy was however associated with high-rates of treatment-related death and exploratory analysis suggested a survival benefit for pre-operative chemoradiotherapy followed by lobectomy when compared to dCRT. In contrast, a closely related trial which was reported in 2015, the ESPATUE trial, found comparable rates of both PFS and overall survival in stage IIIA NSCLC managed with induction chemotherapy followed by either surgery or dCRT [17]. Five-year overall survival was high in both groups, at 44% for those managed with resection and 40% in those treated with dCRT. Historical prospective analyses have provided similar findings. In 2007, the European Organisation for the Research and Treatment of Cancer (EORTC) assessed survival with induction chemotherapy followed by either surgical resection or radiotherapy. No difference in overall survival or progression-free survival was seen [18].

In patients for whom surgical intervention is selected, a key consideration is whether to use pre-operative chemotherapy or CRT. In an attempt to answer this question, the Swiss Group for Clinical Cancer Research compared 117 patients who received pre-operative CRT to 115 who received pre-operative chemotherapy [19]. Both event-free survival and overall survival were comparable for the CRT and chemotherapy groups at 12.8 versus 11.6 months, respectively, for event-free survival, and 37.1 versus 26.2 months for overall survival. Both regimens were relatively well tolerated. For patients receiving CRT, there is good evidence for the use of single-agent cisplatin but not carboplatin when used alongside RT in NSCLC [20, 21].

In the context of N2 disease, the number of mediastinal node stations involved influences prognosis. Several studies have shown that single-station N2 disease is associated with a longer survival (from 40 to 67% in the absence of concomitant pN1 disease) [22–24]. The timing of administration of chemotherapy in relation to surgery in patients with N2 disease is a current point of debate. In the United States, most centres prefer to use chemotherapy in the adjuvant setting whilst in Europe upfront surgery is often used in those cases with single-station non-bulky N2 disease [25, 26].

Regarding the extent of lung resection, pneumonectomy is associated with poorer outcomes than parenchymal-sparing procedures. Lobectomy and sleeve resection are consequently preferred and there remains a lack of consensus on the role of pneumonectomy in N2 disease [27].

### Adjuvant Chemotherapy in Resectable Disease

Adjuvant chemotherapy, broadly defined as chemotherapy administered after surgery in order to reduce the risk of recurrence, is recommended in completely resected stage II and III disease. As discussed earlier, there is in addition some debate about its role in the most advanced stage I cases. It is ordinarily commenced within 8 weeks of surgery, though it can be considered more than 10 weeks post-surgery in suitable patients [28]. A considerable appeal of adjuvant chemotherapy in NSCLC is in attempting to reduce rates of distant metastatic spread, such as to the brain, liver and adrenal gland. Whilst there is now good evidence for the utility of adjuvant chemotherapy in NSCLC, a meta-analysis of early trials undertaken during the 1990s suggested that the use of alkylating-agents was detrimental to overall survival [6]. In contrast, in the same meta-analysis, the use of cisplatin resulted in an apparent survival benefit of 5% at 5 years. There now exists considerable randomised evidence for the benefit of adjuvant Cisplatin in NSCLC, with a subsequent meta-analysis from the Lung Adjuvant Cisplatin Evaluation (LACE) confirming a 5-year benefit with adjuvant chemotherapy of 5.4% [8]. Interestingly, the LACE analysis identified a cumulative cisplatin dose of > 300 mg/m^2^ to be a key prognostic factor and four cycles of cisplatin are therefore generally administered.

The recently published outcomes of the PACIFIC trial suggest that immunotherapy with checkpoint inhibitors may play a significant role in the management of stage III NSCLC [29]. In this study, use of the checkpoint inhibitor durvalumab following dCRT in stage III dCRT resulted in greater median progression-free survival when compared with placebo (16.8 vs. 5.6 months). Overall survival data are awaited, whilst other ongoing trials of checkpoint inhibitors, such as PEARLS (NCT02504372), are seeking to determine whether checkpoint inhibitors have a role following surgical resection.

Despite their promise in the setting of metastatic disease, there is as yet insufficient evidence to conclude on a role for epidermal growth factor receptor (EGFR) inhibitors in locally advanced NSCLC. In the double-blind, phase III RADIANT trial, adjuvant Erlotinib failed to prolong DFS compared with placebo in patients with an EGFR-expressing tumour [30]. Genotype-driven therapy is however under further exploration in both the US ALCHEMIST (NCT02193282) and the Chinese ADJUVANT (NCT01405079) trials. Interim results from the latter were presented at ASCO 2017 and demonstrate significantly greater DFS with the EGFR-inhibitor Gefitinib when compared with standard chemotherapy alone [31].

### Unresectable IIIA and IIIB Disease

In contrast to resectable disease, the management of unresectable stage III NSCLC with definitive chemoradiotherapy is now well standardised and has a clear underlying evidence base. The RTOG 7301 trial determined 60 Gy to be a potentially curative dose of RT in inoperable disease [32]. Local recurrence was nevertheless an issue, prompting early trials in which a significant survival advantage was seen following the addition of induction chemotherapy to definitive RT [33, 34]. As reflected in a meta-analysis of individual patient data, several phase III trials have subsequently shown a benefit from concurrent, rather than sequential, CRT [35]. For those unwilling to undergo or precluded from chemotherapy, accelerated RT may confer improved outcomes [36].

### Superior Sulcus Tumours

Superior sulcus tumours represent an important subgroup for which available evidence indicates that concurrent CRT prior to definitive surgery delivers superior outcomes. The recommendation for the use of this management approach arises from a phase II multicentre trial coordinated by the Southwest Oncology Group [37]. Good long-term survival has subsequently been reported with this approach [38]. The efficacy and safety of a trimodality approach has also been shown in a Japan Clinical Oncology Group phase II trial [39].

### Evidence Gap

Optimal therapy for surgically resectable stage III disease remains debatable and currently the choice of treatment reflects each individual patient’s disease characteristics and the expertise and opinion of the thoracic multi-disciplinary team. Further prospective, ideally randomised, clinical trials are required to determine whether pre-operative chemoradiotherapy followed by surgery, pre-operative chemotherapy followed by surgery ± PORT or definitive chemoradiotherapy is the optimal treatment for resectable stage III NSCLC. Given the results of the PACIFIC trial, the results of adjuvant and neo-adjuvant chemotherapy plus immunotherapy are keenly awaited.

## Oligometastatic Disease: A Subgroup of Stage IV Disease

Platinum-based palliative cytotoxic chemotherapy forms the mainstay of treatment for the 30–50% of patients with NSCLC who present with metastases; common sites for which include the brain, bone, adrenals, liver and lung [40]. Additional therapeutic options to reduce the rate of disease progression include targeted inhibition of anaplastic lymphoma kinase (ALK) and EGFR, in addition to a raft of immune-checkpoint inhibitors. A small number of patients undergo palliative RT and, rarely, surgery for symptomatic relief. Median survival in stage IV NSCLC nevertheless remains low at approximately 8–11 months [40].

Oligometastatic disease is increasingly recognised in a subset of patients with stage IV NSCLC. As first described by Hellman and Weichselbaum in 1995, the oligometastatic state is one in which patients have indolent metastatic disease typically limited to three-to-five sites [41•]. There is as yet no consensus in NSCLC as to the maximum number of metastases that would be classified as oligometastatic disease. However, improvements in surgery and an increasing ability to deliver high-dose, targeted RT via hypofractionated and stereotactic approaches provide mechanisms through which achieving local control of multiple tumour sites without incurring excess morbidity is increasingly possible [42].

An important distinction in oligometastatic disease is whether metastatic lesions are identified at disease diagnosis or following an initial disease-free period, respectively, termed synchronous and metachronous disease. The proportion of patients presenting with synchronous disease is likely to rise as the widespread use of increasingly advanced imaging results in the identification of previously occult metastases [42]. Metachronous disease arises in the context of the isolated progression of one or a small number of metastases after initial treatment. In approximately 60% of patients with relapsed NSCLC, metastases recur in sites already known to have been affected by disease [43]. This supports the presence of an intermediate biological state in which NSCLCs have only limited metastatic potential.

The revision of the M1b staging category into a single metastatic lesion in one organ site (M1b) and multiple metastatic lesions (M1c) within the 8th Edition of the TNM staging system highlights the growing interest in a more radical approach to disseminated NSCLC. This was underpinned by the IASLC’s recognition of comparatively better outcomes in patients with a single extra-thoracic metastasis compared with multiple metastases, either within one organ or across a variety of organs [13••]. However, with the exception of specific isolated extra-thoracic metastases such as intracranial tumours, there is as yet little reliable evidence to guide the use of consolidative therapies in oligometastatic disease and this approach remains unproven [42].

### The Current Evidence Base

Following the closure of a number of prospective studies due to poor accrual, the first multicentre phase 2 randomised controlled trial (RCT) analysing local consolidative treatment (LCT) in oligometastatic NSCLC was reported by Gomez et al. in 2016 [44••]. Using progression-free survival (PFS) as the primary endpoint measure, this trial compared LCT consisting of CRT, RT or resection, with or without maintenance chemotherapy, to maintenance chemotherapy alone. Enrolled patients had a diagnosis of metastatic cancer with up to three metastases and had received standard first-line systemic therapy consisting of either cytotoxic chemotherapy or targeted EGFR/ALK inhibition with no disease progression prior to randomisation. Of the 49 patients enrolled, only three had metachronous metastases. Promisingly, this trial was terminated early by the Data Safety Monitoring Committee at a median follow-up time of 12.4 months after a significantly greater PFS was shown on interim analysis for LCT compared with standard care (11.9 vs. 3.9 months, respectively; HR 0.35), with no difference seen in the incidence of adverse events. Interestingly, a delay was seen in the rate of progression to new metastatic lesions, suggesting a potential systemic benefit from LCT.

These results are however exploratory and must be interpreted with caution [45]. It is not for instance clear whether the presence of up to three metastatic sites truly represents the indolent disease that is thought to characterise the oligometastatic state. The use of PFS as an endpoint is in addition controversial given that whilst LCT would be expected to directly impact on the rate of the progression of known metastatic sites, it is not known whether this translates into a difference in overall survival.

This provocative phase two analysis was underpinned by a number of small prospective and retrospective studies that have suggested that aggressive local control provides benefit in limited metastatic disease. Underling the heterogeneity of these studies, a 2013 systematic review by Ashworth et al. of 2176 patients with oligometastatic NSCLC treated with locally ablative treatments across 49 studies identified significant variation in both median overall survival (5.9–52.0 months) and PFS (4.5–23.7 months) [46]. This is likely to be a result both of the inherent limitations of retrospective and small single-arm prospective analyses, and of variability in treatment approach across existing studies.

In contrast to the significant benefit from LCT identified by Gomez et al., reported outcomes in previous prospective single-arm phase II studies of oligometastatic disease have for the most part been underwhelming. In a 2002 phase II analysis of 23 patients with NSCLC with no greater than N1 involvement and an isolated synchronous site of metastatic disease, Downey et al. trialled surgical resection following induction chemotherapy with Mitomycin C, Vinblastine and Cisplatin [47]. The chemotherapy regimen was poorly tolerated and at 11 (range 1–104) months, median overall survival failed to significantly differ from that seen in historical cohorts. A marginal improvement in overall survival was identified in a more recent prospective phase II study of forty synchronous oligometastatic NSCLC patients, each of whom had fewer than five metastatic sites at primary diagnosis [48]. Treatment options included surgery or RT and systemic therapy was not mandated. Median overall survival in this cohort reached 13.5 months, with a PFS of 12.1 months, though in the absence of a direct comparator cohort the significance of these figures is unclear. Importantly, however, the treatment approach was well tolerated by patients. A similar PFS of 11.2 months was reported by Collen et al. in 2014 [49]. In this study, patients were treated with induction chemotherapy followed by SBRT delivered at a dose of 50 Gy in 10 fractions to both the primary tumour and metastatic locations in 26 patients with five or fewer oligometastatic lesions. The resultant median overall survival of 23 months was greater than that reported previously.

There is in addition-specific evidence for the management of isolated brain, adrenal or lung metastases. Brain metastases are commonly seen in NSCLC, where in the synchronous setting radical treatment using stereotactic radiosurgery (SRS) is associated with a significantly improved overall survival of over 24 months, which is comparable to that of unresectable stage III disease [50]. Solitary metastatic spread to the adrenal may occur in up to one in five patients with metastatic NSCLC. Laparoscopic adrenalectomy has evolved as the standard of care for M1b adrenal disease, though its use is based predominantly on small retrospective series [51, 52]. Similar local control and overall survival rates have however been reported in retrospective analyses of the use of SABR in place of surgery [53]. Both surgery and SBRT have also been reported to be of benefit to the local control of liver oligometastases, though these reports are small and retrospective, and it is not clear to what extent any gain in local control translates to an appreciable improvement in overall survival.

### Specific Treatment Modalities

Surgery has an established role in the management of oligometastatic disease and surgical metastasectomy has been reported in up to 55% of oligometastatic cancers. [54] Alternative treatment modalities include stereotactic RT and thermal ablative techniques directed using interventional radiology, such as cryoablation and radiofrequency ablation (RFA). Effective case selection is increasingly supported by a growing body of evidence relating to prognosticators in oligometastatic disease but there is as yet no direct comparison of alternative treatment modalities.

### Surgery

The surgical approach to oligometastatic disease varies according to the extent and site of metastases. In the context of lung metastases, sublobar resection may deliver reduced toxicity compared with lobectomy or pneumonectomy [55]. Both open and less invasive laparoscopic surgery appear safe and efficacious for adrenal metastases [51]. A 2014 single-centre retrospective analysis of 99 patients treated with metastasectomy for a solitary metastasis reported 5-year survival of 38% [56]. Overall outcomes were superior in patients without mediastinal lymph node involvement, and in those with a pulmonary rather than extra-thoracic metastasis. Interestingly, Johnson et al. have supported a positive role for surgery in mediastinal nodal (N2)-negative disease, reporting promising 5-year survival of 58% in N2-negative patients managed with metastasectomy [57]. Outcomes in surgical series must however be interpreted with caution given that they are likely to be affected by significant selection bias. A number of patients are also precluded from surgery, either through poor performance status or technical difficulty of the location of the metastasis.

### Radiotherapy

The advent of precisely delivered, hypofractionated RT has provided an additional mechanism through which local control of tumour metastases may be obtained. Typically referred to as SABR or SBRT, this high-dose image-guided stereotactic RT is characterised by the delivery of high tumour-doses of RT with a steep-fall off in dose to normal tissues, usually supported by image-guided delivery that accommodates for differences in patient positioning and tumour movement during the ventilatory cycle to limit toxicity to normal tissues [58]. The successful application of SABR/SBRT to oligometastatic disease has been reported both within the context of multi-site extracranial oligometastatic spread and for isolated metastases [59, 60]. However, a recent meta-analysis reported on the use of stereotactic RT in only 15% of patients with oligometastatic disease, though this may reflect the recent development of this technology [46].

A number of prospective trials are ongoing in this area. The Stereotactic Ablative Radiotherapy for Oligometastatic Non-small Cell Lung Cancer (SARON: NCT02417662) randomised controlled trial commenced recruitment in early 2016 and will determine the impact on overall survival of RT (both conventional and SABR) used alongside conventional chemotherapy in the first-line treatment of synchronous oligometastatic disease. In contrast, the phase II/III Conventional Care Versus Radioablation for Extracranial Metastases (CORE: NCT02759783) trial will evaluate SABR used in metachronous oligometastatic disease arising from NSCLC, breast cancer or prostate cancer. In the same setting, the Stereotactic Ablative Radiotherapy for Comprehensive Treatment of Oligometastatic Tumours (SABR-COMET: NCT01446744), which has now closed to recruitment will review the impact of SABR applied to metastatic lesions on both overall survival and quality of life.

### Thermal Ablative Therapy

An alternative approach to the management of oligometastatic disease involves the use of thermal ablative therapies such as radio frequency ablation—RFA (alternatively termed image-guided thermal ablation; IGTA), cryoablation and microwave ablation (MWA). RFA is the most extensively evaluated of these techniques and functions by inducing coagulation necrosis of the lung parenchyma. Much of the research to date has focussed on the use of RFA in medically inoperable patients, often in early NSCLC. The safety and efficacy of RFA in exerting local control of lung tumours have been confirmed in two trials of early NSCLC. In the RAPTURE study, overall survival for patients with high risk disease was 48% at 2 years [61]. This compared with 69.8% in the American College of Surgeons Oncology Group (ACOSOG) Z4033 trial [62]. In both studies, smaller tumour size and a better performance status were associated with superior outcomes. The extra-thoracic use of RFA in NSCLC has is in addition been reported, with high rates of local control seen following treatment of both liver and adrenal lesions [63]. There nevertheless remains a lack of high-level evidence to suggest an impact on survival following RFA and its use is dependent on adequate healthy tissue reserve and the absence of a vascular heat sink.

MWA is increasingly used and has potential advantages over RFA, including the ability to produce a larger volume of necrosis with higher temperatures, shorter treatment time and better penetration in lung tissue. However, high-quality evidence for microwave ablation is lacking and the LUMIRA small randomised controlled trial of lung RFA versus microwave ablation in 52 patients with stage IV disease showed no difference in survival but less pain and more tumour size reduction with MWA [64].

A number of other interventional radiology-guided techniques have been proposed to be of benefit in oligometastatic NSCLC, including chemical ablation, focussed ultrasound ablation and irreversible electroporation. A discussion of these techniques is beyond the scope of this article.

### Combined Treatment

It is probable on the basis of the evidence presented here that future strategies to treat oligometastatic disease will employ surgery, RT and focal interventional-radiology-guided ablative techniques in a manner dependent on the patient’s comorbidities and the location of their metastases. Many studies to date have combined these interventions with chemotherapy and targeted inhibition of EGFR and ALK, which have to-date been the standard of care for stage IV NSCLC. Immunotherapy is an evolving area in lung cancer and is likely to make a significant impact in stage IV disease. Interestingly, a potentially exploitable abscopal effect has been suggested for the combined use of RT with immunotherapy, meaning that radiotherapy to a single metastatic site may lead to regression of other distant sites [65].

### Prognostic Factors

A number of studies have attempted to quantify prognostic factors for both overall survival and PFS in oligometastatic NSCLC using existing retrospective and prospective single-arm phase II studies. A recent hypothesis-generating analysis of 39,910 patients enrolled in the United States Surveillance, Epidemiology and End Results (SEER) database suggested that patients with isolated bone or brain metastases demonstrated comparatively better overall survival than those with metastases to the contralateral lung or to the liver [66].

In their 2013 analysis, Ashworth et al. identified nodal involvement and definitive treatment of the primary tumour as highly significant prognosticators for overall survival [46]. Support for the importance of aggressive thoracic therapy (ATT) to control the primary lesion in synchronous oligometastatic NSCLC has been provided by Li et al. in a systematic review of relevant controlled trials. Concluding on 668 patients, 227 (34%) of whom received ATT, the authors report a significant improvement in OS (HR 0.48) with ATT with survival at one and 4 years of 74.9 and 12.6% following ATT, compared with 32.3 and 2.0% without [67].

In a subsequent individual patient data meta-analysis published a year later, Ashworth et al. analysed a total of 757 NSCLC patients with up to five metastases, the majority of whom (62.3%) received surgery to metastases with the remainder managed with RT [68]. Generating a risk-model, the authors identified nodal involvement and synchronous, as oppose to metachronous, metastases as predictive of worse overall survival. This has been supported by a number of retrospective analyses and it is increasingly accepted that synchronous metastases are likely to reflect more aggressive disease and therefore portend a negative outcome [52, 69, 70]. The literature is not however universally supportive of a disparity in outcome. In a 2001 analysis of 43 patients with an isolated adrenal gland metastasis managed with surgery, Porte and colleagues report a median survival of 11 months with no significant difference in outcomes between metachronous and synchronous presentation [71]. Similarly, a single-centre retrospective analysis of 75 patients found no difference in overall survival or PFS between synchronous and metachronous disease [72].

### Evidence Gap

Evidence for a distinct oligometastatic state in which improved outcomes can be achieved remains minimal and there is as yet no consensus definition for oligometastatic lung cancer. Whilst there is supporting evidence for the aggressive management of isolated metastases, the use of consolidative therapy for multiple metastases remains unproven and existing studies are generally retrospective, include only limited number of patients and are affected by immortal time bias. Further analysis is required to assess both whether aggressive management of oligometastatic NSCLC results in improved outcomes and to define a role for surgery, RT and local ablative therapies. Combinations of ablative techniques with systemic therapy, including immunotherapy and genotype-targeted treatments, also require assessment.

## Conclusions

Evolution of new RT technologies, improved surgical technique and a plethora of interventional-radiology-guided ablative therapies is widening the choice of available treatment modalities to patients with NSCLC. In the setting of resectable locally advanced disease and the oligometastatic state, there is a growing need for randomised comparison of the available treatment modalities to guide both treatment and patient selection.
